# Nutlin-3a Nanodisks Induce p53 Stabilization and Apoptosis in a Subset of Cultured Glioblastoma Cells

**DOI:** 10.4172/2157-7439.1000454

**Published:** 2017-08-23

**Authors:** A Krishnamoorthy, A Witkowski, RO Ryan

**Affiliations:** 1 Department of Nutritional Sciences and Toxicology, University of California Berkeley, Berkeley, CA, USA; 2 Children’s Hospital Oakland Research Institute, 5700 Martin Luther King Jr. Way, Oakland CA, USA; 3 Department of Biochemistry and Molecular Biology, University of Nevada, Reno, NV, USA

**Keywords:** Nutlin-3a, Nanodisk, Apoptosis, Glioblastoma, p53, MDM2

## Abstract

Nanodisks (ND) are ternary complexes of phospholipid, one or more hydrophobic bioactive agents and an apolipoprotein scaffold. These nanoscale assemblies are organized as a disk-shaped lipid bilayer whose perimeter is stabilized by an apolipoprotein scaffold. Solubilization of hydrophobic bioactive agents is achieved by their integration into the ND lipid milieu. When the cis-imidazoline, nutlin-3a, was incubated with phosphatidylcholine and apolipoprotein A-I, it was conferred with aqueous solubility as judged by spectroscopic analysis. Nondenaturing polyacrylamide gel electrophoresis yielded evidence of a homogeneous population of ND particles ~9 nm in diameter. Gel filtration chromatography experiments revealed the association of nutlin-3a with ND is reversible. Biological activity of nutlin-3a ND was examined in three distinct glioblastoma cell lines, U87MG, SF763 and SF767. Incubation of U87MG cells with nutlin-3a ND induced concentration-dependent cell growth arrest and apoptosis. SF763 cells demonstrated modest cell growth arrest only at high concentrations of nutlin-3a ND and no apoptosis. SF767 cells were unaffected by nutlin-3a ND. Immunoblot analysis revealed nutlin-3a ND induced time-dependent stabilization of the master tumor suppressor, p53, and up regulation of the E3 ubiquitin ligase, murine double minute 2 in U87MG cells, but not the other glioma cell lines. The nanoscale size of the formulation particles, their facile assembly and nutlin-3a solubilization capability suggest ND represent a potentially useful vehicle for in vivo administration of this anti-tumor agent

## Introduction

**Figure 1:**
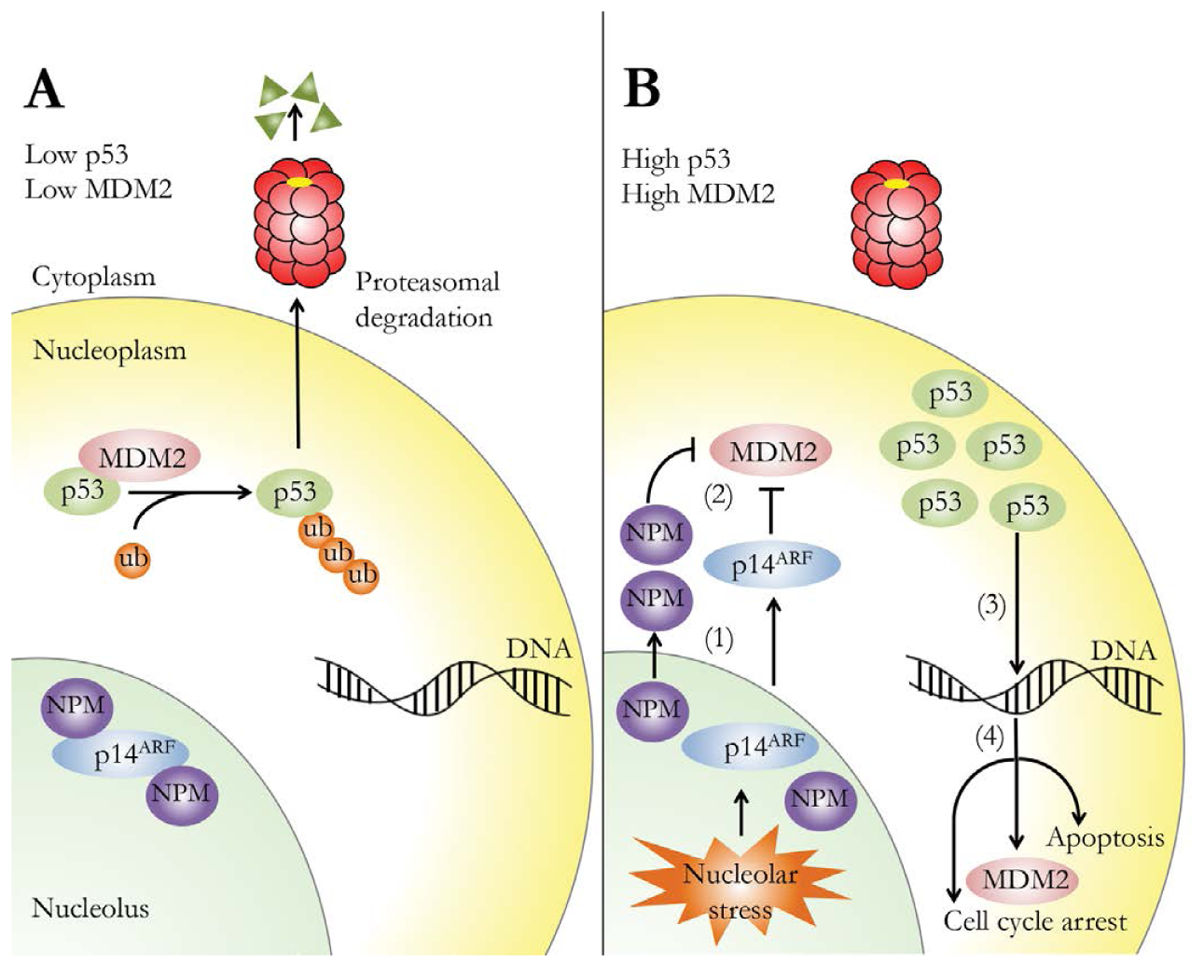
The response of p53, MDM2, p14^ARF^ and NPM to nucleolar stress. In healthy cells (panel A), p53 is targeted for proteasomal degradation by MDM2, and p14^ARF^ binds NPM in the nucleolus. In response to nucleolar stress (panel B), p14^ARF^ and/or NPM migrate from the nucleolus to the nucleoplasm (1) and binds MDM2, inhibiting its E3 ubiquitin ligase activity (2). As a result, levels of p53 rise, promoting its function as a transcriptional activator (3) of genes of cell cycle arrest, MDM2 expression and apoptosis (4).

Healthy cells maintain low levels of the tumor suppressor protein, p53 through the action of the E3 ubiquitin ligase, murine double minute 2 (MDM2). Continuous ubiquitinylation of newly expressed p53 by MDM2 targets it for degradation by the 26S proteasome [1] as shown in Figure 1. At the same time, other regulatory proteins, including p14ARF and nucleophosmin (NPM) are sequestered in the nucleolus as part of larger protein complexes. In response to cellular stress (e.g. oncogene activation, genotoxic drugs or disruption of rRNA synthesis by chemotherapeutic drugs), p14ARF and/or NPM redistribute to the nucleoplasm where they function as inhibitors of MDM2 ubiquitin ligase activity [2–5]. As a result, p53 levels rise, promoting transactivation of genes involved in cell cycle arrest and DNA damage repair [6]. An important gene target of p53 is MDM2 itself, creating an auto-regulatory feedback loop that limits the duration of the stress response [7–9]. If the damage is irreparable, p53 stabilization leads to activation of an apoptotic response [10]. Given its central role in the regulation of cell fate, many cancers are caused by mutations in the p53 pathway [8,11].

Astrocytic gliomas are one of the most common intracranial malignant tumors, with four classification grades (I-IV). The most severe form (Grade IV), referred to as glioblastoma multiforme (GBM), is one of the most aggressive and least effectively treated solid tumors in humans [12]. Close to 78% of GBMs have a disrupted p53 pathway, including deletion of p14ARF, overexpression of MDM2 or mislocalization of p53 to the cytoplasm [13]. The current standard of care for newly diagnosed patients with GBM involves surgical resection, with concurrent radiotherapy and administration of the cytotoxic alkylating agent, temozolomide [14,15]. Despite this aggressive and invasive treatment strategy, nearly all GBMs recur within 15 months, resulting in high rates of morbidity and mortality [16].

In 2004, a family of chemically related cis-imidazoline based compounds, termed nutlins, were shown to inhibit MDM2 activity [17]. X-ray crystallography of a nutlin-2-MDM2 complex revealed it binds to a tryptophan-rich pocket in MDM2 previously identified as a p53 binding site [18]. Thus, it is postulated that nutlins compete with, or displace, p53 from MDM2 [19]. It may be anticipated that pharmacological inhibition of MDM2’s ubiquitin ligase activity by nutlin represents a plausible therapeutic approach in tumors that possess a wild type (WT) p53 pathway [20–22]. However, a general problem with nutlins relates to their intrinsic hydrophobicity. Poor water solubility poses practical problems with respect to mode of administration, bioavailability, toxicity and transit across the blood brain barrier [23].

One approach to overcoming these obstacles is to solubilize nutlin in a delivery vehicle. Toward this end, nanoscale disk-like miniature membranes, termed nanodisks (ND), have been documented to solubilize various hydrophobic bioactive agents including amphotericin B [24], all trans retinoic acid [25] and curcumin [26]. ND selfassembly occurs upon incubation of specific phospholipids with members of the class of exchangeable apolipoproteins [27]. Based on its structural properties, solubility profile and potent biological activity, nutlin-3a has been identified as a candidate for integration into ND. In the current study, nutlin-3a ND were formulated and shown to elicit variable effects on cultured GBM cells, depending on their p53 pathway status.

## Material and Methods

### Reagents

Nutlin-3a (the most active member of this inhibitor class) was obtained from Cayman Chemical (Ann Arbor, MI) and dissolved in dimethylformamide (DMF). Dimyristoylphosphatidylcholine (DMPC) was obtained from Avanti Polar Lipids Inc. (Alabaster, AL). Bicinchoninic acid (BCA), bovine serum albumin (BSA) and GelCode Blue stain reagent were purchased from Thermo Fisher Scientific. Mouse anti-p53 (clone DO-1) and mouse anti-MDM2 were from Sigma Aldrich. Anti-GAPDH was from EMD Millipore (San Diego, CA). Horseradish Peroxidase (HRP) conjugated goat anti-mouse IgG secondary antibody was from Vector laboratories (Burlingame, CA). WesternBright ECL HRP substrate was from Advansta (Menlo Park, CA). Halt Protease Inhibitor cocktail was from Thermo Scientific (Rockford, IL). Rabbit anti-p14ARF was purchased from Novus Biologicals (Littleton, CO). Mouse anti-NPM was purchased from Abcam (Cambridge, United Kingdom). FITC conjugated goat polyclonal anti-rabbit IgG secondary antibody (probe for p14ARF) was purchased from Abcam (Cambridge, United Kingdom) and Texas Red conjugated goat monoclonal anti-mouse IgG secondary antibody (probe for NPM) was purchased from Santa Cruz Biotechnology (Dallas, TX). Recombinant WT human apolipoprotein (apo) A-I and tryptophan (W)-null variant apoA-I (the four W residues in apoA-I were replaced by phenylalanine) were expressed in *E. coli* and isolated as described previously [28]. The GBM cell lines, SF763 and SF767, were kindly provided by Dr. Eleanor Blakely (Lawrence Berkeley National Laboratory). U87MG cells were purchased from the UC Berkeley Cell Culture Facility. Cells were maintained in high glucose DMEM (Thermo Scientific Hyclone, South Logan, UT) supplemented with 10% fetal bovine serum, 100 U/ml penicillin and 100 μg/ml streptomycin. Cells were cultured at 37°C in a humidified atmosphere of 5% CO2 and 95% air. CellTiter 96 Non-Radioactive Cell Proliferation (MTT) assay kit was purchased from Promega (Madison, WI). FITC - Annexin V Apoptosis kit was purchased from Invitrogen (Carlsbad, CA).

### Nutlin-3a ND formulation

Five mg DMPC was dissolved in chloroform/methanol (3:1 v/v) and dried under a stream of N2 gas, forming a thin film on the vessel wall. Residual organic solvent was removed under vacuum. The prepared lipid was dispersed in 0.5 ml phosphate buffered saline (PBS; 20 mM sodium phosphate, 150 mM sodium chloride, pH 7.4) and 400 μg nutlin-3a (from a 20 mg/ml stock solution) added. Following this, 2 mg apoA-I (from a 4 mg/ml stock solution in PBS) was added and the sample (1 ml final volume) was bath sonicated between 22°C and 25°C for approximately 45 min. The sonication step induced the turbid sample to clarify, indicating that the complexes of apolipoprotein and phospholipid (i.e. ND) had formed. The sample was centrifuged at 10,000 rpm for 10 min to remove unincorporated material and filter sterilized (0.22 μm). Control ND (termed empty ND) was prepared in a similar manner except nutlin-3a was omitted from the reaction mix.

### UV/Vis absorbance spectroscopy

Absorbance spectroscopy was performed on a Perkin-Elmer Lambda 20 spectrophotometer. Samples were scanned from 260–350 nm.

### Non-denaturing polyacrylamide gel electrophoresis (PAGE)

Samples (~6 μg protein) were electrophoresed on 4–20% acrylamide slab gels at a constant 150 V for 2 h at 22°C and stained with GelCode Blue.

### Gel filtration

A sterile filtered sample (200 μl) of nutlin-3a ND was applied to a Zorbax GF-250 column equilibrated in PBS containing additionally 0.15 M NaCl. Chromatography was performed on a Perkin-Elmer Series 200 System at a flow rate of 1 ml/min. Fractions (0.5 ml) were collected over a period of 30 min. For comparison, a sample containing empty ND was applied to the column.

### Cell viability assay

Cells were plated in 96-well culture plates at 10,000 cells per well and allowed to attach overnight. Subsequently, the cells were replenished with fresh medium supplemented with specified concentrations of nutlin-3a ND or a corresponding amount of empty ND. The range of nutlin-3a concentrations tested (20 μM to 100 μM nutlin-3a) was limited by the amount of nutlin-3a that could be solubilized in ND. Furthermore, in preliminary experiments on nutlin-3a ND stability, it was determined that storage of nutlin-3a ND for two weeks at 4°C resulted in decreased biological activity toward U87MG cells. To avoid this, all experiments reported were performed using freshly prepared nutlin-3a ND. After 24 h incubation with ND, cell proliferation assays were performed. Briefly, cells were incubated with MTT (3-[4,5-dimethylthiazol-2-yl]-2,5-diphenyltetrazolium bromide) for 4 h at 37°C. Viable cells converted the tetrazolium into purple colored formazan crystals that were solubilized upon incubation for 1 h at 37°C, prior to absorbance measurement at 570 and 650 nm.

### Apoptosis assay

Cells were plated in 12-well culture plates at 0.5 × 106 cells per well and allowed to attach overnight. Subsequently, the cells were replenished with fresh medium supplemented with specified concentrations of nutlin-3a ND or a corresponding amount of empty ND. Twenty-four h after treatment, apoptosis was measured by flow cytometry. Briefly, cells were scraped from the plate, washed with ice cold PBS and re-suspended in 106 μl binding buffer containing 0.5% BSA, 5 μl FITC-annexin V and 1 μl propidium iodide and incubated, shielded from light for 30 min at room temperature. Cells were pelleted to remove unbound dye and re-suspended in binding buffer containing 0.5% BSA. Flow cytometry measurements were obtained on BD LSR Fortessa and data analyzed using FlowJo v10 software.

### Immunoblot analysis

Cells were plated in 12-well culture plates at 0.5 × 106 cells per well and allowed to attach overnight. Cells were then incubated with fresh medium supplemented with 50 μM nutlin-3a ND, a corresponding amount of empty ND or PBS. At indicated time points, cells were lysed with RIPA buffer (50 mM Tris, 150 mM NaCl, 0.1% SDS, 0.5% sodium deoxycholate, 1% Triton X-100 and 1× Halt Protease Inhibitor cocktail). Protein concentrations were determined and 10 μg proteins from each lysate were separated on a 4% to 20% acrylamide SDS gel, transferred to a PVDF membrane and blocked with 5% (w/v) non-fat milk. Target proteins were visualized using the ECL detection system after incubation with anti-p53 (1:1000), anti-MDM2 (1:200) or anti-GAPDH (1:2000) antibodies. Goat anti-mouse HRP-conjugated secondary antibody was used at (1:5000) dilution.

### Confocal fluorescence microscopy

U87MG and SF767 cells were plated in 12-well culture plates with coverslips at 0.5 × 106 cells per well and allowed to attach overnight. Cells were incubated with 50 μM nutlin-3a (as ND) for 4 h at 37°C. Coverslips were washed, the cells were fixed with 4% paraformaldehyde for 15 min on ice and washed with PBS containing 1% BSA. Cells were permeabilized with 0.1% Triton X-100 in PBS for 10 min at room temperature followed by 3 washes with PBS containing 1% BSA. Fixed cells were incubated with anti-NPM (1:100) and anti-p14ARF (1:100) for 2 h at room temperature, then washed and incubated again with Texas Red conjugated goat anti-mouse IgG (1:200) for NPM detection and FITC conjugated goat anti-rabbit IgG (1:200) for p14ARF detection. Finally, cells were stained with Hoeschst 33342 (1:2000) in PBS for 10 min at room temperature and washed before mounting with Vectashield. Coverslips on slides were allowed to dry overnight before image acquisition on a Zeiss LSM710 confocal microscope.

### Statistical analysis

Statistical significance between groups was calculated using the two-tailed Student’s t-test. Data are presented as mean ± standard error (SE) of three independent experiments performed in triplicate, with p-values < 0.05 considered significant.

## Results

### Nutlin-3a ND formulation

**Figure 2:**
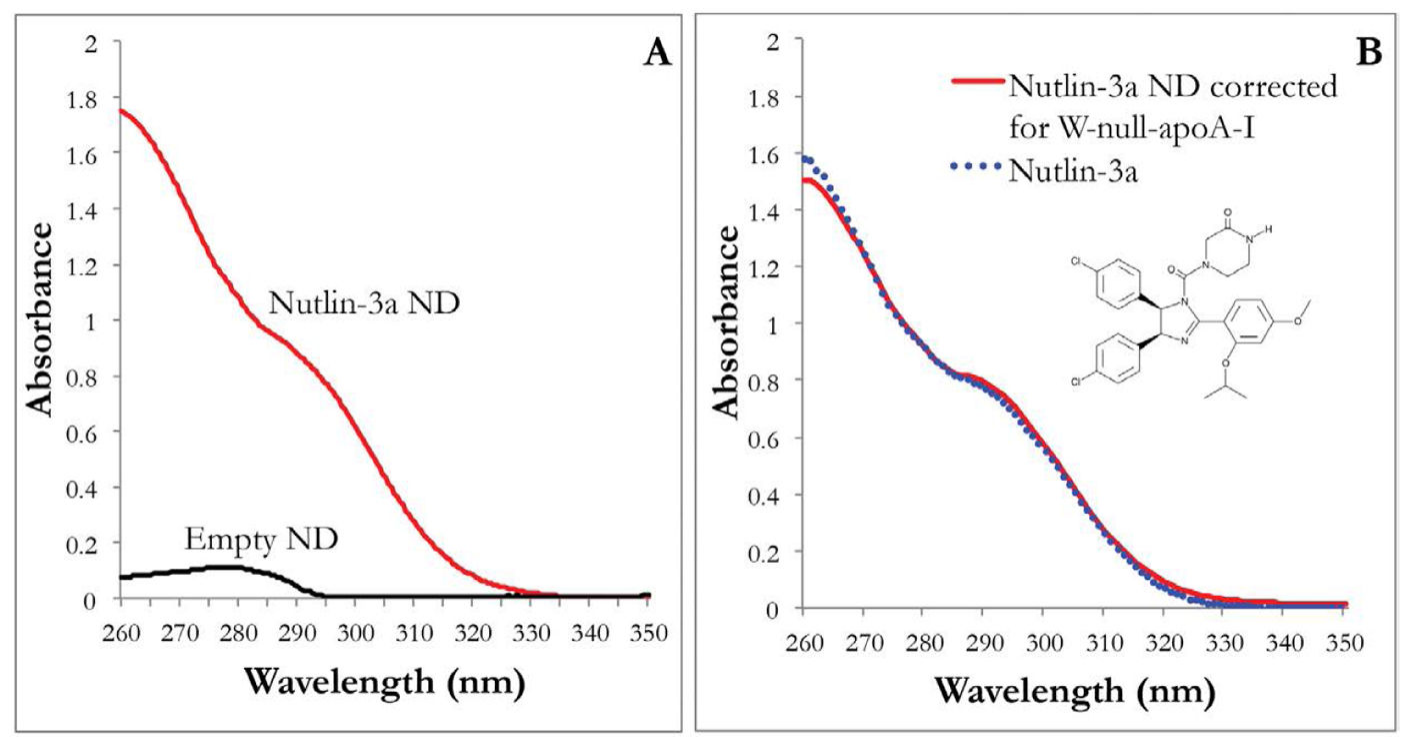
UV-Visible absorbance of nutlin-3a ND, empty ND and nutlin-3a. (A) 400 μg nutlin-3a in nutlin-3a ND or equivalent volume of DMF in empty ND (both prepared using W-null apoA-I as ND scaffold protein) in 1 ml final volume of PBS, were scanned. (B) Absorbance of 400 μg nutlin-3a (in DMSO) and nutlin-3a ND (in DMSO) (spectrum of empty ND (in DMSO) was subtracted from nutlin-3a ND (in DMSO) to account for absorbance from W-null apoA-I. Spectrum of DMSO was subtracted from spectrum of nutlin-3a).

**Figure 3:**
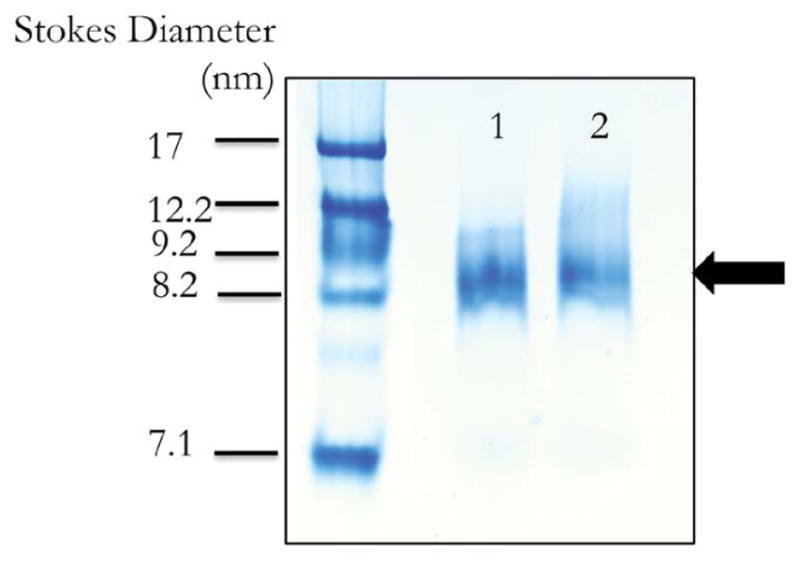
Nondenaturing PAGE of ND preparations. A 4–20% nondenaturing PAGE gel was electrophoresed at 150V for 2 h. Lane 1) empty ND; Lane 2) nutlin-3a ND. Stokes diameter was estimated from the migration of known molecular markers run on same gel. Black arrow depicts the band corresponding to ND.

Nutlin-3a is poorly soluble in aqueous media yet fully miscible in organic solvents. In preliminary experiments, nutlin-3a in DMSO was observed to absorb light in the range of 260–320 nm, a property that was used to monitor solubilization of nutlin-3a in ND. When nutlin-3a was incubated with DMPC and recombinant WT apoA-I, although the solution appearance indicated nutlin-3a ND had formed, spectral overlap from the four tryptophan (W) residues in apoA-I (absorbance max=280 nm) interfered with spectroscopic quantitation of nutlin-3a solubilization efficiency. To circumvent this, a W-null-apoA-I variant was generated and employed in lieu of WT apoA-I as ND scaffold. As with experiments using WT apoA-I, the sample transitioned from an opaque, turbid suspension into a clear solution, indicating ND formation. Following centrifugation and sterile filtration (0.22 μm), a UV-visible absorbance spectrum was recorded. In contrast to empty ND (no nutlin-3a) prepared with W-null-A-I, nutlin-3a ND gave rise to an absorbance spectrum consistent with nutlin-3a solubilization (Figure 2A). The minor absorbance observed in the empty ND sample can be attributed to the 7 tyrosine residues in W-null-apoA-I. After subtracting the absorbance contribution from W-null-apo-A-I, the UV-vis absorbance pattern of nutlin-3a ND was found to superimpose with a spectrum of the same amount of nutlin-3a in organic solvent (Figure 2B) indicating that, at this concentration, nutlin-3a efficiently incorporates into ND. Nondenaturing gradient PAGE of nutlin-3a ND revealed a population of particles with a diameter ~9 nm, similar to the size of control empty ND (Figure 3). Upon gel filtration chromatography both nutlin-3a ND and empty ND eluted between 8 and 12 min post-injection. However, nutlin-3a was not detected in the elution fractions containing ND, indicating nutlin-3a association with ND is reversible without destruction of the ND vehicle.

### Effect of nutlin-3a ND on cell viability

**Figure 4:**
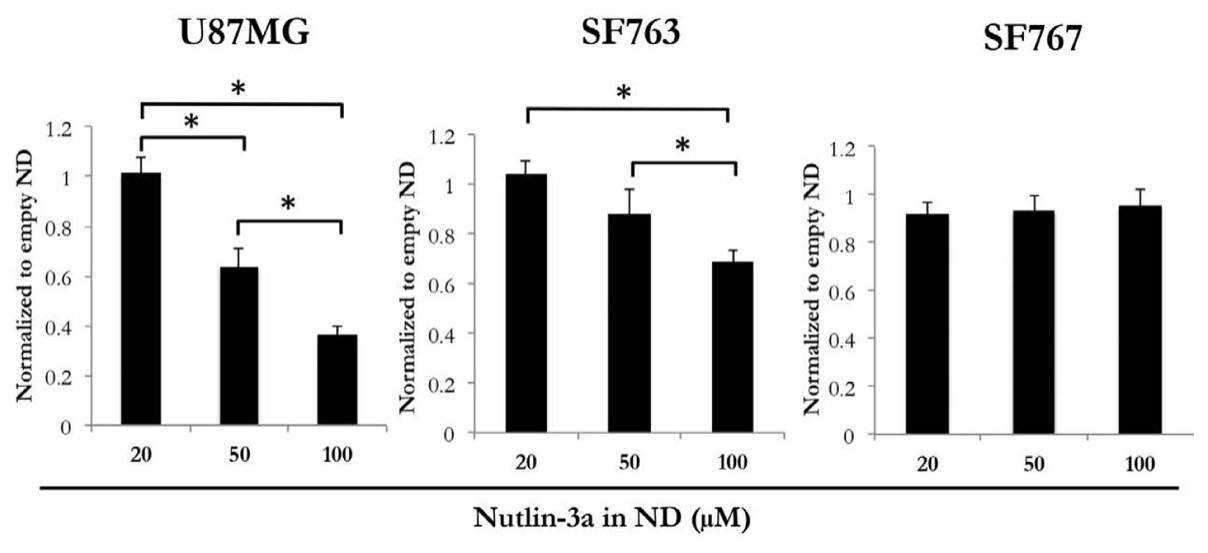
The effect of nutlin-3a on the viability of cultured glioma cells. Three GBM cell lines (U87MG, SF763 and SF767) were incubated with nutlin-3a ND or empty ND at indicated concentrations. The cells were processed as described in Materials and Methods and cell viability measured by the MTT assay. Samples treated with nutlin-3a ND were compared to samples treated with a corresponding amount of empty ND. Values reported are the mean ± SE of 3 independent experiments performed in triplicate. *p<0.05.

To assess the biological activity of nutlin-3a ND, three distinct GBM cell lines were examined. U87MG cells possess a WT p53 pathway [29,30] while SF763 cells harbor a mutant p53 [29,31] and SF767 cells have a different mutation that results in constitutively elevated MDM2 protein levels [32,33]. Each of these GBM cell lines were incubated with nutlin-3a ND or empty ND for 24 h, followed by MTT assay of cell viability as described in Methods. Of the three cell lines examined, U87MG cells displayed the greatest response to nutlin-3a ND (Figure 4). SF763 cell viability was modestly affected at the highest concentration of nutlin-3a ND while SF767 cell viability was largely unaffected by incubation with nutlin-3a ND.

### Nutlin-3a ND induced apoptosis

**Figure 5:**
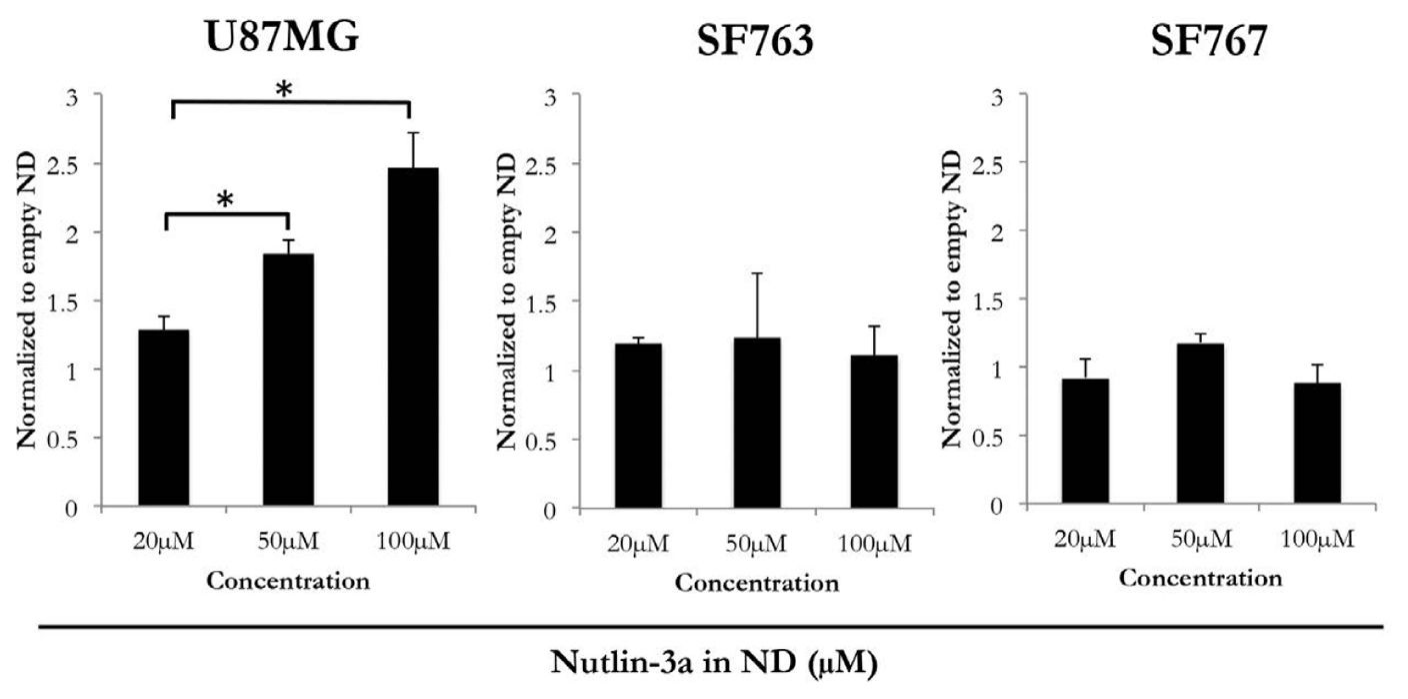
Effect of nutlin-3a ND on GBM cell apoptosis. Three GBM cell lines (U87MG, SF763 and SF767) were incubated with nutlin-3a ND or empty ND at the indicated concentrations. After 24 h, the cells were processed as described in Materials and Methods. The cells were incubated with FITC labeled annexin-V and propidium iodide and after washing, subjected to flow cytometry. Cells treated with nutlin-3a ND were compared to cells treated with empty ND. Values reported are the mean ± SE of 3 independent experiments. ^*^p<0.05.

To evaluate the ability of nutlin-3a ND to induce cell death via apoptosis, U87MG, SF763 and SF767 cells were incubated with increasing concentrations of nutlin-3a (as ND) or a corresponding amount of empty ND for 24 h followed by flow cytometry analysis of apoptosis based on FITC-annexin V binding and propidium iodide fluorescence intensity. Complementing the cell viability results, U87MG cells displayed a pronounced apoptotic response when treated with nutlin-3a ND (Figure 5) while SF763 and SF767 were unresponsive over this nutlin-3a concentration range.

### Effect of nutlin-3a on p53 and MDM2 levels

**Figure 6:**
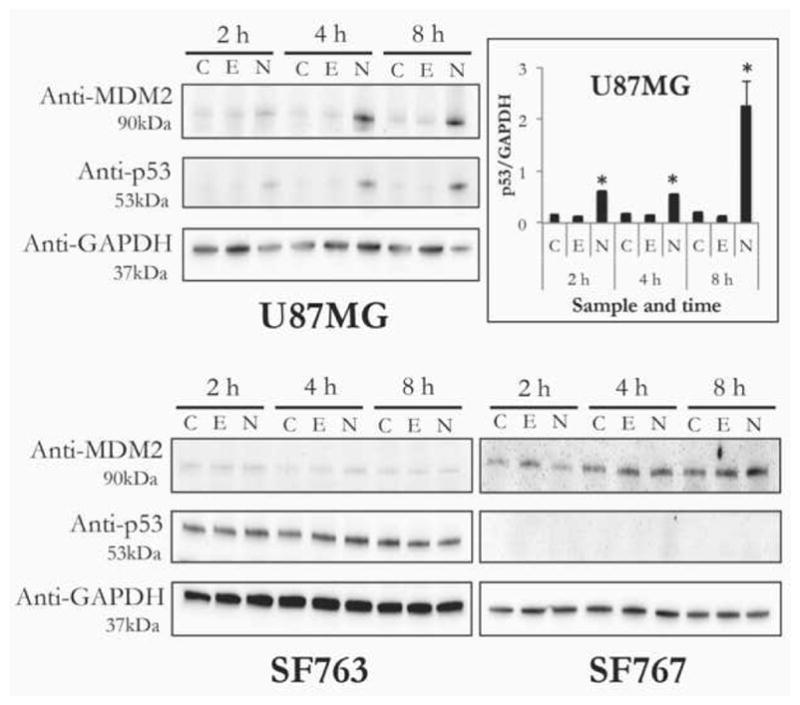
Effect of nutlin-3a ND on cellular levels of p53 and MDM2. U87MG, SF763 and SF767 cells were incubated with PBS, empty ND or 50 μM nutlin-3a (as ND) for 2, 4 and 8 h. At indicated time points, cells were harvested, lysed and 10 μg cell protein separated by SDS PAGE, immunoblotted and probed with antibodies directed against MDM2, p53 and GAPDH. Lane assignments: C, PBS treated control; E, empty ND; N, nutlin-3a ND. Bar graph represents densitometric analysis of p53 band intensity in U87MG cells, normalized to internal loading control (i.e. GAPDH). Values reported are the mean ± SE of 3 independent experiments. * p<0.05 versus empty ND and PBS treated cells.

To further investigate the effects of nutlin-3a on cell viability and apoptosis, immunoblot analysis of p53 and MDM2 was performed. Each of the three GBM cell lines were incubated with 50 μM nutlin-3a (as ND), empty ND or buffer and harvested after 2, 4 and 8 h. Following cell lysis, 10 μg cell protein was separated by SDS-PAGE, immunoblotted and probed with antibodies against p53, MDM2 and GAPDH. As shown in Figure 6, compared to incubations with buffer (labeled C) or empty ND (E), U87MG cells treated with nutlin-3a ND (N) showed a time dependent increase in p53 and MDM2 content. In the case of SF763 cells, MDM2 was barely detectable and p53 levels were elevated under all incubation conditions. Conversely, in SF767 cells, MDM2 levels were elevated regardless of the incubation condition and p53 was undetectable.

### Effect of nutlin-3a ND on p14ARF and NPM distribution

**Figure 7:**
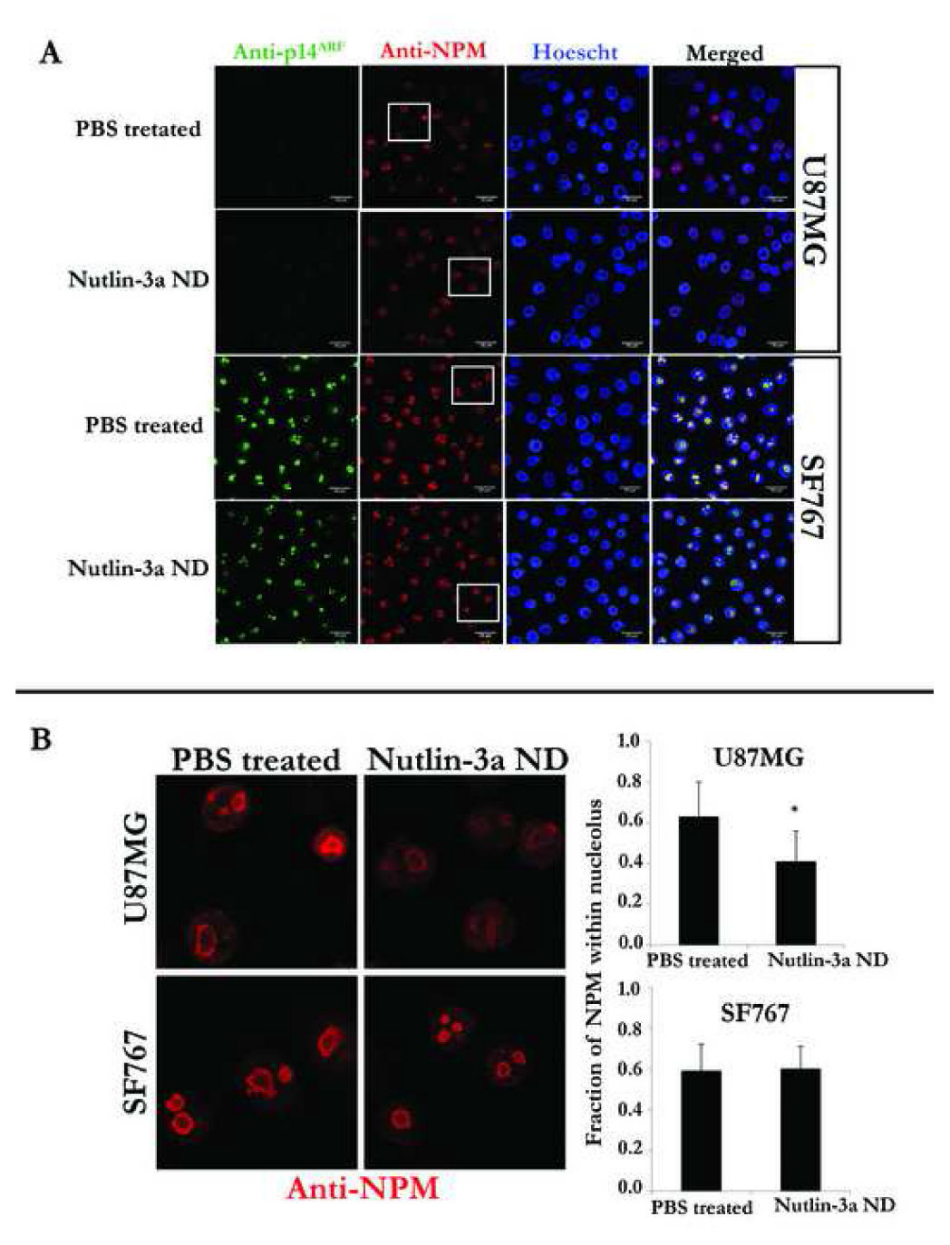
Effect of nutlin-3a ND on the sub nuclear localization of p14ARF and NPM. Panel A) U87MG and SF767 cells grown on cover slips were incubated with PBS or 50 μM nutlin-3a (as ND) for 4 h at 37°C. Anti-p14ARF and anti-NPM was added to the fixed cells and secondary antibodies tagged with FITC (p14ARF) or Texas Red (NPM) was used for detection. Cell nuclei were stained with Hoescht. Scale bar 20 μm. Panel B) Higher magnification images of cells bordered by white boxes in the anti-NPM region of panel A. Bar graph represents densitometric analysis of immunofluorescence intensity processed in grayscale mode using ImageJ software. Values reported are mean ± SE of 15 or more cells in each group examined for NPM localization. *p<0.05 versus PBS treated. Background readings were corrected using the formula: Corrected Total Cell Fluorescence (CTCF)=Total Fluorescence – (Area of selected cell x Mean fluorescence of background readings). Fraction of NPM within nucleolus=(CTCF nucleolus)/(CTCF nucleus). Integrated density values were used for calculations.

To investigate the effect of nutlin-3a ND on the cellular localization of p53 pathway regulatory proteins p14ARF and NPM, U87MG and SF767 cells were incubated with 50 μM nutlin-3a (as ND) for 4 h and analyzed by confocal fluorescence microscopy. In U87MG cells, no p14ARF was detected in control or nutlin-3a ND treated cells (Figure 7A). Whereas NPM localized to the nucleolus in U87MG cells incubated with PBS, it displayed a more diffuse nuclear distribution pattern in cells treated with nutlin-3a ND (Figure 7B). In SF767 cells, p14ARF and NPM remained localized in the nucleolus in the absence and presence of nutlin-3a ND.

## Discussion

Nutlins have been used to treat different types of cancer, usually in combination with chemotherapeutic drugs, anti-mitotic agents and radiation [19,34–39]. However, little progress has been made in terms of their use to treat glioblastomas. One reason for this is poor solubility of nutlin-3a in aqueous media, a property that poses a challenge for administration and likely affects bioavailability. In an effort to confer water solubility, nutlin-3a was incorporated into ND, particles previously shown to function as a transport vehicle for hydrophobic bioactive compounds [27]. Nutlin-3a was originally identified based on its ability to induce apoptosis and cell cycle arrest by activating upstream regulators of the tumor suppressor protein, p53 [17]. Thus, in cells possessing a WT p53 pathway, nutlin-3a administration induces cell cycle arrest and/or apoptosis. To examine the effect of nutlin-3a ND, three GBM cell lines were studied. Among these grade IV gliomas, U87MG cells possess a functional p53/MDM2 pathway while the other two cell lines harbor mutations that result in constitutively elevated p53 (SF763 cells) or MDM2 (SF767 cells) protein levels [29,30,32]. Upon incubation with nutlin-3a ND, U87MG cells were most responsive in terms of compromised cell viability and increased number of apoptotic cells. At the same time, SF763 cells showed reduced cell viability only at high doses of nutlin-3a ND and SF767 cells were unaffected. In cells with a mutant p53 protein (i.e. SF763), p53 levels were elevated to begin with and nutlin-3a ND did not induce any change in p53 content. SF767 cells failed to show a biological effect despite reports that it possesses a functional p53 [33]. It is conceivable, however, that the constitutively high MDM2 protein levels in this cell line could not be overcome by the nutlin-3a concentrations examined such that residual E3 ubiquitin ligase activity of MDM2 promoted continued degradation of p53.

Although U87MG cells are deficient in p14ARF [40], they responded to nutlin-3a ND by increasing p53 protein levels. The observation that nutlin-3a ND induced NPM redistribution from nucleolus to nucleoplasm suggests that either nutlin-3a is capable of directly activating a cellular stress response or NPM redistribution is a consequence, rather than a cause, of the cellular stress response induced by nutlin-3a ND. Considering the variable response of the 3 GBM cell lines to nutlin-3a ND, the data indicate that detailed knowledge of a patients’ p53 pathway status is key to assessing whether nutlin-3a treatment may be of therapeutic benefit.

At present, surgical resection followed by radiation and chemotherapy is the standard of care for GBM. Many drug delivery systems have been investigated including targeted delivery via overexpressed receptors not present (or present at low levels) in normal tissue, convection enhanced delivery and intra-arterial delivery with blood brain barrier disruption [41]. Despite advances in treatment options, median patient survival remains an abysmal 8–22 months, most likely due to the highly infiltrative nature of these tumors [42]. In order to eliminate remnant tumor cells, it is conceivable that, following surgical resection, insertion of a biologically inert implant containing nutlin-3a ND into the surgical cavity may be effective [43]. Release/ diffusion of nutlin-3a ND from the implant over time is anticipated to promote apoptosis of remnant tumor cells, thereby improving patient survival. Taken together, the findings reported herein support further evaluation of nutlin-3a ND as a therapy option for GBMs that possess an intact p53 pathway.

## References

[1] Michael D, Oren M (2003). The p53-Mdm2 module and the ubiquitin system. Semin Cancer Biol.

[2] Brodska B, Holoubek A, Otevrelova P, Kuzelová K (2016). Low-Dose Actinomycin-D Induces Redistribution of Wild-Type and Mutated Nucleophosmin Followed by Cell Death in Leukemic Cells. J Cell Biochem.

[3] Di Matteo A, Franceschini M, Chiarella S, Rocchio S, Travaglini-Allocatelli C (2016). Molecules that target nucleophosmin for cancer treatment: an update. Oncotarget.

[4] Sherr CJ (2006). Divorcing ARF and p53: an unsettled case. Nat Rev Cancer.

[5] Suzuki A, Kogo R, Kawahara K, Sasaki M, Nishio (2012). A new PICTure of nucleolar stress. Cancer Sci.

[6] Lane DP (1992). Cancer. p53 guardian of the genome. Nature.

[7] Barone G, Tweddle DA, Shohet JM, Chesler L, Moreno L (2014). MDM2-p53 interaction in paediatric solid tumours: preclinical rationale, biomarkers and resistance. Curr Drug Targets.

[8] Wasylishen AR, Lozano G (2016). Attenuating the p53 Pathway in Human Cancers: Many Means to the Same End. Cold Spring Harb Perspect Med.

[9] Zhao Y, Yu H, Hu W (2014). The regulation of MDM2 oncogene and its impact on human cancers. Acta Biochim Biophys Sin.

[10] Roos WP, Kaina B (2006). DNA damage-induced cell death by apoptosis. Trends Mol Med.

[11] Vijayakumaran R, Tan KH, Miranda PJ, Haupt S, Haupt Y (2015). Regulation of Mutant p53 Protein Expression. Front Oncol.

[12] Rao JS (2003). Molecular mechanisms of glioma invasiveness: the role of proteases. Nat Rev Cancer.

[13] Cancer Genome Atlas Research Network (2008). Comprehensive genomic characterization defines human glioblastoma genes and core pathways. Nature.

[14] Fritz L, Dirven L, Reijneveld J, Koekkoek J, Stiggelbout A (2016). Advance Care Planning in Glioblastoma Patients. Cancers (Basel).

[15] Hau E, Shen H, Clark C, Graham PH, Koh ES (2016). The evolving roles and controversies of radiotherapy in the treatment of glioblastoma. J Med Radiat Sci.

[16] Ramakrishna R, Pisapia D (2015). Recent Molecular Advances in Our Understanding of Glioma. Cureus.

[17] Vassilev LT, Vu BT, Graves B, Carvajal D, Podlaski F (2004). In vivo activation of the p53 pathway by small-molecule antagonists of MDM2. Science.

[18] Kussie PH, Gorina S, Marechal V, Elenbaas B, Moreau J (1996). Structure of the MDM2 oncoprotein bound to the p53 tumor suppressor transactivation domain. Science.

[19] Laurie NA, Donovan SL, Shih CS, Zhang J, Mills N (2006). Inactivation of the p53 pathway in retinoblastoma. Nature.

[20] England B, Huang T, Karsy M (2013). Current understanding of the role and targeting of tumor suppressor p53 in glioblastoma multiforme. Tumour Biol.

[21] Khoo KH, Hoe KK, Verma CS, Lane DP (2014). Drugging the p53 pathway: understanding the route to clinical efficacy. Nat Rev Drug Discov.

[22] Villalonga-Planells R, Coll-Mulet L, Martínez-Soler F, Castaño E, Acebes JJ (2011). Activation of p53 by nutlin-3a induces apoptosis and cellular senescence in human glioblastoma multiforme. PLoS One.

[23] Burgess A, Chia KM, Haupt S, Thomas D, Haupt Y (2016). Clinical Overview of MDM2/X-Targeted Therapies. Front Oncol.

[24] Oda MN, Hargreaves PL, Beckstead JA, Redmond KA, van Antwerpen R (2006). Reconstituted high density lipoprotein enriched with the polyene antibiotic amphotericin. B J Lipid Res.

[25] Redmond KA, Nguyen TS, Ryan RO (2007). All-trans-retinoic acid nanodisks. Int J Pharm.

[26] Ghosh M, Singh ATK, Xu W, Sulchek T, Gordon LI (2011). Curcumin nanodisks: formulation and characterization. Nanomedicine.

[27] Ryan RO (2010). Nanobiotechnology applications of reconstituted high density lipoprotein. J Nanobiotechnology.

[28] Ryan RO, Forte TM, Oda MN (2003). Optimized bacterial expression of human apolipoprotein A-I. Protein Expr Purif.

[29] Geoerger B, Vassal G, Opolon P, Dirven CM, Morizet J (2004). Oncolytic activity of p53-expressing conditionally replicative adenovirus AdDelta24-p53 against human malignant glioma. Cancer Res.

[30] Cerrato JA, Yung WK, Liu TJ (2001). Introduction of mutant p53 into a wildtype p53-expressing glioma cell line confers sensitivity to Ad-p53-induced apoptosis. Neuro Oncol.

[31] Van BVW, Van DDPB, Grill J, Pinedo HM, Gerritsen WR (2002). Conditionally replicative adenovirus expressing p53 exhibits enhanced oncolytic potency. Cancer Res.

[32] Blough MD, Zlatescu MC, Cairncross JG (2007). O6-methylguanine-DNA methyltransferase regulation by p53 in astrocytic cells. Cancer Res.

[33] Trentin GA, He Y, Wu DC, Tang D, Adcock MR (2004). Identification of a hTid-1 mutation which sensitizes gliomas to apoptosis. FEBS Lett.

[34] Cao C, Shinohara ET, Subhawong TK, Geng L, Kim KW (2006). Radiosensitization of lung cancer by nutlin, an inhibitor of murine double minute 2. Mol Cancer Ther.

[35] Carvajal D, Tovar C, Yang H, Vu BT, Heimbrook DC (2005). Activation of p53 by MDM2 antagonists can protect proliferating cells from mitotic inhibitors. Cancer Res.

[36] Coll-Mulet L, Iglesias-Serret D, Santidrián AF, Cosialls AM, de Frias M (2006). MDM2 antagonists activate p53 and synergize with genotoxic drugs in B-cell chronic lymphocytic leukemia cells. Blood.

[37] Drakos E, Thomaides A, Medeiros LJ, Li J, Leventaki V (2007). Inhibition of p53-murine double minute 2 interaction by nutlin-3A stabilizes p53 and induces cell cycle arrest and apoptosis in Hodgkin lymphoma. Clin Cancer Res.

[38] Kojima K, Konopleva M, McQueen T, O Brien S, Plunkett W (2006). Mdm2 inhibitor Nutlin-3a induces p53-mediated apoptosis by transcription-dependent and transcription-independent mechanisms and may overcome Atm-mediated resistance to fludarabine in chronic lymphocytic leukemia. Blood.

[39] Secchiero P, Barbarotto E, Tiribelli M, Zerbinati C, di Iasio MG (2006). Functional integrity of the p53-mediated apoptotic pathway induced by the nongenotoxic agent nutlin-3 in B-cell chronic lymphocytic leukemia (B-CLL). Blood.

[40] Ishii N, Maier D, Merlo A, Tada M, Sawamura Y (1999). Frequent Co-Alterations of TP53, p16/CDKN2A, p14ARF, PTEN Tumor Suppressor Genes in Human Glioma Cell Lines. Brain Pathol.

[41] Laquintana V, Trapani A, Denora N, Wang F, Gallo JM (2009). New strategies to deliver anticancer drugs to brain tumors. Expert Opin Drug Deliv.

[42] Young RM, Jamshidi A, Davis G, Sherman JH (2015). Current trends in the surgical management and treatment of adult glioblastoma. Ann Transl Med.

[43] Bastiancich C, Danhier P, Préat V, Danhier F (2016). Anticancer drug-loaded hydrogels as drug delivery systems for the local treatment of glioblastoma. J Control Release.

